# Editorial: Eating behavior and chronic diseases: research evidence from population studies, volume II

**DOI:** 10.3389/fnut.2026.1831436

**Published:** 2026-04-08

**Authors:** Fei Xu, Ling Zeng, Zumin Shi

**Affiliations:** 1 Nanjing Medical University Affiliated Nanjing Municipal Center for Disease Control and Prevention, Nanjing, China; 2 School of Population Health, The University of New South Wales, Sydney, NSW, Australia; 3 College of Health Sciences, QU Health, Qatar University, Doha, Qatar

**Keywords:** chronic disease, dietary pattern, eating behavior, nutritional epidemiology, population-based evidence

Non-communicable diseases (NCDs) have a broad spectrum of unfavorable chronic health conditions (1). Usually, NCDs are mentioned as these four main types of chronic disease—cardiovascular disease (heart disease, and stroke), cancer, diabetes, and chronic lung disease (1). Collectively, they impose substantial disease burden worldwide, accounting for approximately 74% of all the global deaths presently (1). However, besides these four commonly-mentioned NCDs, obesity, osteoporosis, gout, and even some gastrointestinal disorders are also within the broad concept of NCD (1). It has been well-documented that NCDs share five major risk factors: tobacco use, physical inactivity, the harmful use of alcohol, unhealthy diets, and air pollution (1, 2). Fortunately, these risk factors are modifiable (1, 2). Clearly, for unhealthy diets and alcohol drinking, they are eating behavior related risk factors of NCDs.

For population-level NCDs prevention, it is of lasting public health significance to improve evidence-based healthy eating behaviors. This highlights the necessity of population-based epidemiological research on human nutrition and eating behaviors. Critically, based on epidemiological research approaches, studies have been becoming a continuously hot and popular topic on community-based interventions of eating behavior, associations between selected eating behaviors and NCDs, and dynamical/periodical investigation/surveillance of specific eating behavior or dietary patterns among overall- or/and sub-populations across the world. Therefore, the Research Topic of *Eating Behavior and Chronic Diseases: Research Evidence from Population Studies* is welcome and makes contributions to expanding the research area in healthy eating and NCDs.

Obesity, for both adults and adolescents, is a big public health problem worldwide, as it is not only a chronic condition itself but also a driving factor for the main NCDs (3). Using the most recent data from ten Eastern European countries—Belarus, Bulgaria, Czechia, Hungary, Poland, the Republic of Moldova, Romania, the Russian Federation,

Slovakia, and Ukraine (4), the study of *Energy Intake and Dietary Fiber as Principal Determinants of Obesity in Eastern Europe, 2010–2*022 (Siminiuc et al.) reported the population-level associations of dietary energy availability and dietary fiber intake with obesity and overweight prevalence among adults in Eastern Europe during 2010–2022. It was examined that energy availability was positively, while dietary fiber was negatively, associated with both adulthood obesity and overweight in eastern European countries. Moreover, in another paper, *Association between Fruit Intake and Abdominal Adiposity in 1707 Randomly Selected U.S. Children* (Tucker), fruit intake was investigated to be in negative relation to central obesity among children in USA based on NHANES 2011–2016 data. These two studies reinforced that low-energy-densed and high-fiber-densed foods are likely of help for weight control for either adults or children.

Cardiovascular disease and diabetes are two of the four common main NCDs defined by World Health Organization (1). Therefore, to investigate the association of eating behavior with them collectively is of particular public health importance. A meta-analysis based on prospective data, *Coffee and Tea Consumption and Cardiovascular Disease and All-Cause and Cause-Specific Mortality in Individuals with Diabetes Mellitus: a Meta-Analysis of Prospective Observational Studies* (Ding et al.), addressed that daily coffee or tea consumption was associated with lower risks of cardiovascular disease incidence and death among diabetic individuals. The interesting findings in this study have significant implications, suggesting that coffee and/or tea consumption may be favorable for diabetes patients to reduce risks of developing cardiovascular diseases.

Metabolic syndrome (MetS), a complex cardio-metabolic condition, is typically defined by co-existence of at least three of the five components: abdominal obesity, raised fasting blood glucose, increased blood pressure, elevated triglyceride, and/or lowered high-density lipoprotein cholesterol (5). Evidence from nutritional epidemiological studies may be more informative for understanding associations of eating behaviors with MetS as well as its components. In the investigation, *Relationship between Skipping Breakfast and Metabolic Syndrome among Adults Aged 35–74 Years: A Cross-Sectional Study in Northwest China, 2018–2020* (Yang et al.), it was observed that breakfast skipping, an unhealthy eating behavior, was significantly associated with MetS among adults in regional China. This study implied an easily practicable potential approach for MetS prevention, breakfast eating, for adults to prevent MetS.

Another common main NCD is chronic obstructive pulmonary disease (COPD), one of chronic respiratory diseases. Relative to classical influencing factors (e.g., tobacco smoke exposure, occupational exposure to dusts, fumes or chemicals, and indoor air pollution, etc.), eating behavior was less investigated regarding the potential association with COPD (6–10). In the paper of *Low-Carbohydrate Diet Score and Chronic Obstructive Pulmonary Disease: a Machine Learning Analysis of NHANES data* (Zhang et al.), it was examined that low-carbohydrate diet was associated with lower odds of COPD among American adults aged 40 years and older. Additionally, findings from the study, *The Association of Vegetable Consumption and Physical Activity with Chronic Obstructive Pulmonary Disease among Community-Dwelling Adults Aged 40 Years or Above in China* (Kang et al.), showed that vegetable intake was negatively linked with COPD among adults aged 40 years or above in regional China. These two studies among adults from USA and China consistently suggest that unhealthy eating behaviors may be a potentially plausible driver of COPD for adults.

In addition to the four commonly-mentioned NCDs, hyperuricemia and gout are alcohol-drinking-associated chronic metabolic diseases (11), also imposing substantial disease burden globally (12, 13). In the study of *Impact of Alcohol Consumption on Hyperuricemia and Gout: a Systematic Review and Meta-Analysis* (Ma et al.), the association of alcohol drinking with hyperuricemia and gout were systematically updated based on meta-analysis of most recent publications. Meaningfully, a significant dose-response relationship was examined in this meta-analysis, confirming the positive link between alcohol drinking and these two conditions, particularly for men.

Moreover, chronic gastric disorder (CGD), a well-established risk factor for gastric cancer, is also within the broad concept of NCDs (14). Previous studies documented inconsistent findings on the relationship between vegetable consumption and the risk of CGD in China, the country with a high prevalence of CGD (15–17). The study, *The Relationship between Vegetable Intake and Chronic Gastric Disorder among Community-dwelling Residents Aged 35 Years and Older in China* (Guan et al.), reported a positive association between vegetable consumption and CGD among more than 38,000 community-dwelling adults aged 35 years and older in regional China. Considering the randomly-selected representative participants with large sample size, and the consistent findings among overall or age-, sex-, location-stratified participants, the positive association of vegetable intake and CGD may hold for general adult population in China.

Monitoring and surveillance of eating behaviors or dietary patterns are the classical research area of nutritional epidemiology study. Periodically updated data on eating behaviors or dietary patterns are crucial for informing time-dependent and population-specific community-based intervention campaigns, and evaluation of intervention effectiveness. The Mediterranean diet (MD) pattern is a widely recognized health-friendly eating behavior (18). There is strong evidence showing that adherence to MD pattern is negatively associated with NCDs (19). Recently, using data collected in 2023 from adults aged 18 years and older in regional Spain, the study, *Adherence to Mediterranean Diet and Physical Activity Practice in General Population: an Intersectional Analysis of Inequalities by Sex and Economic Status* (Reig-García et al.), reported that there were 67.8% adults adhering to MD pattern, and revealed a disparity in the prevalence of adherence between men and women. Another survey implemented in 2023 among community-dwelling residents aged 18 years or above in a typical mega-city of China, *Patterns of Meat and Vegetable Consumption among Community-Dwelling Adults Aged 18 Years and Older in China* (Deng et al.), updated the scenarios of meat and vegetable consumption among adults in China. Based on this study, local adults consumed meat and vegetables at median levels of 700.0 g/wk and 200.0 g/d, respectively, while only 18.1% and 28.9% of residents met the intake levels of meat and vegetable, separately, recommended by Chinese Nutrition Society in 2022 (20). These two studies provide an update of eating behavior among adults in the 1^st^ year after COVID-19 pandemic under the context of traditional Western and Eastern food cultures, separately.

For population-based healthy eating promotion, food-related broadcasting/media content is a potentially helpful option in today's society with widely spreading media-based information. It has been investigated that food-related broadcasting contents may contribute to unhealthy eating behaviors, as they can provide emotional connection and vicarious satisfaction for stimulating eating scenes (21). In the study—*Association between Food-Related Media Content and the Eating Behaviors of Korean Adults according to Household Type* (Yun et al.)—conducted in 2024 among adults aged 20–65 years in South Korea, food-related digital content viewing was examined to be positively associated with two eating behaviors, late-night eating and food-delivery/take-out. These findings suggest that food-related broadcasting/media content may encourage unhealthy eating behaviors for adults. Moreover, it implies that exposure to food-related media should be considered as intervention approaches of healthy eating promotion campaigns.

**Figure 1:**
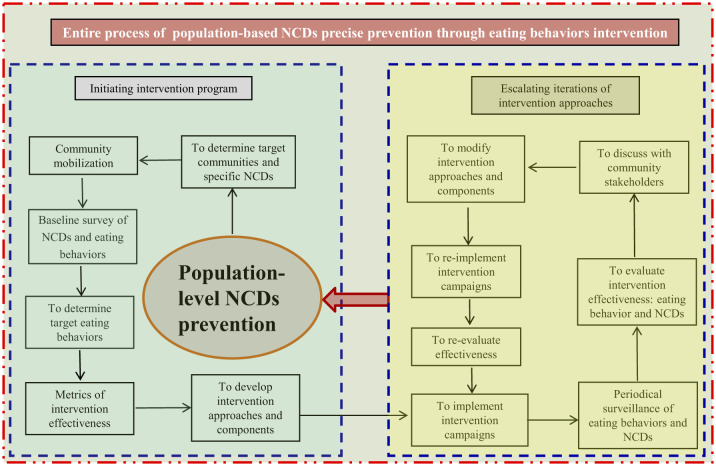
Roadmap for NCDs precise prevention through eating behavior intervention.

Population-based healthy eating interventions are an effective strategy for prevention of eating behavior related NCDs (22–25). However, the precise eating behavior intervention is time-dependent, population- and culture-specific, which calls for periodical surveillance/monitoring of eating behavior and NCDs, consistent and timely evaluation of eating behavior intervention effectiveness, and optimal modification of intervention approaches for target populations. This shall be a dynamically rolling and improving process of intervention, surveillance, evaluation, program modification, and re-intervention for community-based eating-related NCDs prevention campaigns. Such an intervention roadmap of population-level precise prevention of eating-related NCDs was displayed in Figure 1.

The papers included in the Research Topic provided new evidence regarding the associations of eating behaviors with cardiovascular diseases, metabolic syndrome, COPD, obesity, hyperuricemia, gout, and chronic gastric disorder. Moreover, specific eating behavior (meat and vegetable intake) and pattern (Mediterranean diet) as well as association of broadcasting media with eating behaviors were also investigated. These studies add significant values to literature and have public health implications for eating behavior-associated NCD prevention. Human eating behavior is highly subject to age, time, economic status, and environment. This highlights that, for the purpose of community-based precise prevention of NCDs, continuous studies shall be encouraged on associations of specific eating behaviors and selected NCDs, surveillance of population-level eating behaviors, and effective and actionable healthy eating campaigns among economically, and culturally diverse sub-populations worldwide.
